# Pallister-Hall Syndrome Presenting in Adolescence

**DOI:** 10.1155/2019/6845836

**Published:** 2019-03-18

**Authors:** Aria Mahtabfar, Niall Buckley, Susan Murphy, Shabbar Danish, Ian Marshall

**Affiliations:** ^1^Rutgers-Robert Wood Johnson Medical School, 675 Hoes Lane, Piscataway Township, NJ 08854, USA; ^2^Division of Pediatric Hematology and Oncology, Rutgers-Robert Wood Johnson Medical School, Cancer Institute of New Jersey, 195 Little Albany Street, New Brunswick, NJ 08901, USA; ^3^Department of Neurosurgery, Rutgers-Robert Wood Johnson Medical School, Cancer Institute of New Jersey, 195 Little Albany Street, New Brunswick, NJ 08901, USA; ^4^Division of Pediatric Endocrinology, Rutgers-Robert Wood Johnson Medical School, 89 French Street 2nd Floor, New Brunswick, NJ 08901, USA

## Abstract

Pallister-Hall syndrome (PHS) is an extremely rare syndrome of unknown prevalence with autosomal dominant inheritance due to* GLI3 *gene mutations classically characterized by the presence of a hypothalamic hamartoma and polydactyly. Additional diagnostic criteria include bifid epiglottis, imperforate anus, small nails, hypopituitarism, growth hormone deficiency, and genital hypoplasia. It is typically diagnosed in infancy and early childhood, presenting with seizures and/or precocious puberty due to the hypothalamic hamartoma, and with limb anomalies due to central polydactyly. Our patient had presented with polysyndactyly at birth. However, as this is not uncommon in infants and is usually as part of the sporadic, isolated form of polydactyly, no further work up was done. He then presented at age 16 years with a headache and subjective visual changes, with brain imaging revealing a hypothalamic hamartoma. He did not have a history of seizures or central precocious puberty. Genotyping revealed a pathogenic variant affecting the* GLI3 *gene. We encourage all clinicians to consider PHS or an associated syndrome with a clinical finding of polydactyly. Further, as the natural history continues to reveal itself, this patient's presentation provides important new data to the broad phenotypic spectrum of PHS.

## 1. Introduction

Pallister-Hall syndrome (PHS) was first characterized in 6 infants by Hall et al. in 1980 as a neonatally lethal malformation syndrome of hypothalamic hamartoblastoma, postaxial polydactyly, and imperforate anus, along with a constellation of visceral and endocrine abnormalities [1, 2]. Additional sporadic and familial cases were subsequently reported, resulting in a National Institute of Health workshop to further characterize PHS in 1996 [3]. This workshop established minimum diagnostic criteria for PHS that included the presence of both a hypothalamic hamartoma and mesoaxial polydactyly [3]. Afterward, criteria for sub-PHS were established to include 1 of the following: mesoaxial polydactyly (also known as central polydactyly with an additional partial or complete digit involving 2nd, 3rd, or 4th fingers or toes), postaxial polydactyly (additional partial or complete digit located on ulnar margin of the hand, or lateral to 5th toe), oligodactyly (presence of fewer than 5 digits on the hand or foot), or hypothalamic hamartoma; AND 1 of the following: bifid epiglottis, imperforate anus, small nails, hypopituitarism, growth hormone deficiency, or genital hypoplasia [4].

In 1997, Kang et al. identified* GLI3* gene mutations as the cause of PHS [5]. The inheritance is typically autosomal dominant with* de novo* mutations in certain cases [4]. Mutations located outside of the middle third of the same* GLI3* gene result in other syndromes, including Greig cephalopolysyndactyly syndrome [6], isolated polydactyly types A, A/B, and preaxial polydactyly type 4 [7, 8], and acrocallosal syndrome [9].

Endocrine manifestations of hypothalamic hamartomas consist almost exclusively of central precocious puberty (CPP) [10]. Although hypopituitarism, growth hormone deficiency, and genital hypoplasia have been described in PHS, it is not clear whether these are secondary to the hamartoma [10]. Neurologic manifestations of the hamartoma consist of gelastic seizures [11]. In contrast to hypothalamic hamartomas that occur in isolation in nonsyndromic patients, PHS patients are less likely to develop seizures, and if they do, tend to occur later in life, less frequently, and are more easily controlled pharmacologically [12].

In this case report, we describe the presentation at 16 years of age of a male with new onset headaches and temporary vision loss, whose brain imaging revealed a nonenhancing, isointense, hypothalamic mass, on a background of mesoaxial and postaxial polydactyly consistent with PHS.

## 2. Case Presentation

This 16-year-old male presented to the emergency department with new onset headache and visual changes of 2 days duration. The headache was described as sudden in onset, constant in nature, and bitemporal in location. This was associated with development of vision loss 1 day characterized by darkening of his vision, progressing from the superior to the inferior visual fields that lasted approximately 30 minutes with subsequent persistence of blurry vision. Without resolution of his symptoms, he presented to the emergency department.

On questioning, there was no prior history of seizure activity or of precocious puberty, or of genital abnormalities at birth. He had undergone corrective surgery for polydactyly of the left hand at 9 months of age. Polydactyly was also reported in his mother and elder brother.

His neurological examination was benign without abnormalities of extraocular movements, pupillary reflexes, facial motor strength, or sensation; he did not have papilledema. His genitourinary examination was normal and appropriate for age. Inspection of the left hand revealed webbing between the 3rd and 4th digits, with a well-healed scar on the ulnar aspect over the 5th metacarpophalangeal joint.

**Figure 1:**
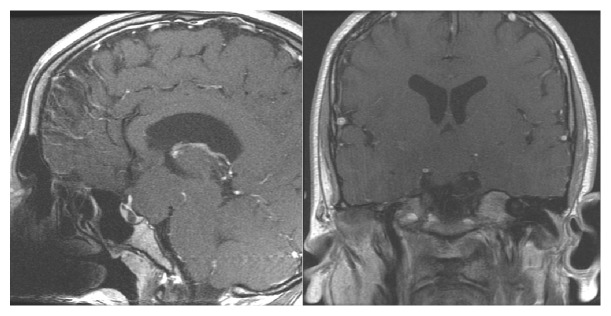
Sagittal and Axial MRI with contrast showing isointense, nonenhancing hypothalamic mass.

CT scan of the head without contrast identified a 30 mm mass in the suprasellar region. Follow-up MRI of the brain with and without contrast confirmed this, identifying a nonenhancing, 30 x 27 x 30 mm mass along the midline of the posterior aspect of the suprasellar cistern contiguous with the posterior floor of the hypothalamus and tuber cinereum (Figure 1). The mass was isointense to the gray matter on all sequences and appeared to produce significant mass effect on the posterior aspect of the optic chiasm and both optic tracts, with anterior displacement of the pituitary stalk.

Hormonal testing revealed intact anterior pituitary gland function. There were no symptoms suggestive of central diabetes insipidus. Serum germ cell markers were negative. Formal visual field testing did not exhibit deficits. Formal otolaryngology evaluation did not reveal a bifid uvula or epiglottis.

**Figure 2:**
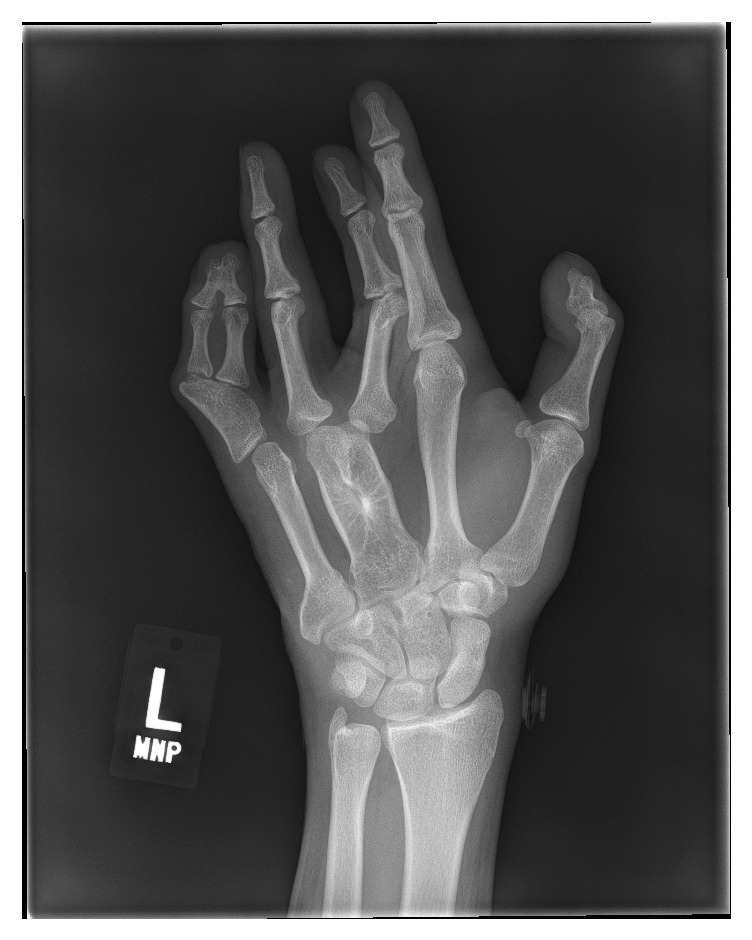
Hand X-ray exhibiting polysyndactyly of the 3rd and 4th metacarpals, and polydactyly of the middle and distal phalanges of the 5th finger. Subluxation of unknown age of the proximal phalanx of the 3rd finger was coincidentally noted.

Radiographs of the left hand revealed several morphologic abnormalities, including partial fusion (syndactyly) of the 3rd and 4th metacarpals, and polydactyly of the middle and distal phalanges of the 5th finger (Figure 2).

While the headache persisted for 24 hours and then resolved with use of analgesics, the visual changes resolved spontaneously within 4 hours of admission. The hypothalamic hamartoma was suggested to be asymptomatic and not the cause for his temporary visual loss. Other etiologies were not identified.

Based on the polysyndactyly of his left hand and MRI findings consistent with a hypothalamic hamartoma, PHS was considered.

While optic nerve decompression via right supraorbital craniotomy was considered because of the acute visual changes, the multidisciplinary team and patient elected for close follow-up without intervention in light of the syndromic presentation, negative formal visual field testing, and historically benign nature of hamartomas.

Subsequent gene sequencing of* GLI3 *performed at GeneDx (Gaithersburg, MD) revealed a heterozygous c.2388delT variant causing a frameshift, starting with codon Leucine 797, changing the amnio acid Leucine to a Tyrosine, and thus creating a premature stop codon at position 12 of the new reading frame, denoted p.Leu797TyrfsX12. This pathogenic variant is predicted to cause loss of normal protein through protein truncation. This variant had also not been previously reported. We were unable to clinically and genetically evaluate his mother and brother. Genetic counseling was offered but declined by the family.

## 3. Discussion

Despite numerous case reports as well as a foundational understanding of limb abnormalities originating from the elucidation of PHS molecular biology, this syndrome is rare with the true incidence and natural history not well established [4].

This case report describes a unique presentation of genetically confirmed PHS in a 16 year old male, as it is typically diagnosed in infancy and early childhood, when the 2 essential features, the hypothalamic hamartoma and mesoaxial polydactyly, may present with seizures and/or precocious puberty, and with limb anomalies, respectively [4]. This patient had presented with meso and postaxial polydactyly as well as syndactyly of the 3rd and 4th metacarpals at birth. Polydactyly is relatively common in infants [13]. Although typically of the sporadic, isolated form [13], polydactyly can also be a feature of about 300 well-characterized syndromic malformations [14]. Clinically, there are no typical features of the polydactyly to suggest that it forms part of a specific syndrome including PHS. The patient then presented at age 16 years with a headache and subjective visual changes. Interestingly, despite transient vision loss, careful visual field examination did not elicit any deficits. Further, despite the relatively large hamartoma size on imaging, and correlation of development of CPP with hamartoma size [12], this patient did not have history of seizures or CPP.

Given the absence of endocrine abnormalities, focal neurologic and visual deficits, with spontaneous resolution of the headache and visual changes, the obvious risks associated with surgical intervention exceeded the expected benefit. Approaches for resection of hypothalamic hamartomas have been explored in the literature. Recommended open surgical techniques include subtemporal approach for hypothalamic hamartomas that are pedunculated or have prepontine components, as well as transcallosal approach for sessile lesions with intraventricular components [15, 16]. Radiosurgery, using Gamma knife surgery, is also being investigated, particularly for nonsurgical candidates [15].

Although rare with approximately only 100 reported cases of PHS [4], it is important for clinicians to consider whether a finding of polydactyly could represent PHS, or an associated syndrome. As the natural history of PHS continues to reveal itself, with more sophisticated geno- and phenotyping, our patient's clinical presentation and genetic analysis makes a significant contribution to the broad and new phenotypic spectrum of PHS.
